# Clinical values of AFP, GPC3 mRNA in peripheral blood for prediction of hepatocellular carcinoma recurrence following OLT: AFP, GPC3 mRNA for prediction of HCC

**Published:** 2011-03-01

**Authors:** Yuliang Wang, Zhongyang Shen, Zhijun Zhu, Ruifa Han, Mingsheng Huai

**Affiliations:** 1 Orient Organ Transplant Center, First Central Clinic Institute of Tianjin Medical University, Key Lab for Critical Care Medicine of the Ministry of Health, Tianjin, China; 2 Tianjin Institute of Urology, Tianjin Medical University, Tianjin, China

**Keywords:** Liver transplantation, Hepatocellular carcinoma, Alpha-fetoprotein, Glypican-3, Recurrence

## Abstract

**Background:**

Hepatocellular carcinoma (HCC) is one of the most common malignancies worldwide. Annually, about 200,000 patients died of HCC in China. Liver transplantation (LT) holds great theoretical appeal in treating HCC. However, the high recurrence rate after transplantation is the most important limiting factor for long-term survival.

**Objectives:**

To assess the value of alpha-fetoprotein (AFP) messenger RNA (mRNA), Glypican-3 (GPC3) mRNA-expressing cells in the peripheral blood (PB) for prediction of HCC recurrence following orthotopic liver transplantation (OLT).

**Patients and Methods:**

29 patients with HCC who underwent OLT with a minimum clinical follow-up of 12 months were included in this retrospective study. We detected AFP mRNA, GPC3 mRNA-expressing cells in the PB by TaqMan real-time reverse transcriptase-polymerase chain reaction (RT-PCR), pre-, intra- and post-operatively. The early recurrence of patients was evaluated.

**Results:**

8 (28%), 15 (52%), and 9 (31%) patients had AFP mRNA detected pre-, intra-, and post-operatively, respectively. With 12 months of follow-up, HCC recurred in 7 (24%) patients. Univariate analysis revealed that positive pre- and post-operative AFP mRNA, TNM stage as well as vascular invasion were significant predictors for the HCC recurrence. Multivariate analysis revealed that being positive for AFP mRNA pre-operatively remained a significant risk factor for HCC recurrence after OLT. GPC3 mRNA was expressed in all PB samples. There was no significant difference in the expression levels of GPC3 mRNA between the HCC and control groups. There were no significant differences in GPC3 mRNA expression values between those patients with and without tumor recurrence.

**Conclusions:**

The pre-operative detection of circulating AFP mRNA-expressing cells could be a useful predictor for HCC recurrence following OLT. GPC3 mRNA-expressing cells in PB seem to have no diagnostic value.

## Background

Hepatocellular carcinoma (HCC) is one of the most common malignancies worldwide, with a global incidence of 500,000 new cases per year; 82% of cases (and deaths) occur in developing countries (with 55% in China) [1][2]. HCC is up to four times more common in men than in women; 60%-90% of these tumors develop in a cirrhotic liver [3]. Each year, about 200,000 patients died of HCC in China. Liver transplantation (LT), which offers the potential to both resect the entire potentially tumor-bearing liver and eliminate the cirrhotic tissues, holds great theoretical appeal in treating HCC. However, recurrence of HCC is a significant cause of mortality after LT [4][5]. Even with the implementation of Milan criteria, recurrence rates have been shown to be 8%-15% in most studies [6][7][8]. The high recurrence rate after transplantation is the most important limiting factor for long-term survival. Establishment of a direct and accurate method to predict tumor recurrence and/or micrometastasis after LT is needed. The human α-fetoprotein (AFP) messenger RNA (mRNA) is generally accepted as a tumor-specific marker. There are some reports to identify AFP mRNA for detecting isolated tumor cells (ITC) in peripheral blood (PB) [9][10]. It sounds theoretically reasonable to consider the detection of circulating HCC cells as being predictive for HCC recurrence after LT. We have established an accurate and sensitive method for the detection of circulating HCC cells by RT-PCR method to quantify AFP mRNA [11]. Glypican-3 (GPC3) is a member of the glypican family of cell-surface heparin-sulfate proteoglycans, which is linked to the cell surface through a glycosylphosphatidylinositol (GPI) anchor [12]. There has been considerable interest in GPC3, because it is markedly overexpressed in a high proportion of HCCs and promotes the growth of HCCs [13][14][15]. Some clinical research studies reported GPC3 overexpression in HCC both at mRNA and protein levels [16][17]. Based on these results, it has been proposed that GPC3 could be used as a serum and histochemical marker for HCC. Despite this clinical interest in GPC3, it is still unclear whether GPC3 mRNA detectable in PB is related to HCC diagnosis and predicts its recurrence.

## Objectives

In the present study, we applied TaqMan real-time RT-PCR to assess the usefulness of detecting AFP mRNA and GPC3 mRNA-expressing cells in the PB for predicting the risk of HCC recurrence after orthotopic liver transplantation (OLT).

## Patients and Methods

### Patients and Sample Collection

Twenty-nine liver transplant recipients with HCC (27 men, 2 women; mean age: 48 years, age range: 37-62 years) who underwent OLT in Orient Organ Transplant Center during 2008 were included in this retrospective study. The diagnosis of HCC was confirmed by pathologic study by experienced pathologists. The criteria for OLT in patients with HCC are absence of extrahepatic malignancies, macroscopic tumor thrombosis, or extrahepatic metastasis of HCC. Of the 29 studied patients, 27 had hepatitis B virus (HBV)-induced cirrhosis, and two patients had hepatitis C virus (HCV)-induced cirrhosis. Twelve patients had received treatment for HCC; treatments included resection, radiotherapy or transcatheter hepatic arterial chemoembolization (TACE) prior to transplantation. The remaining 17 patients had not received any treatments. Clinical characteristics of HCC patients are summarized in Table 1. All recipients had undergone successful OLT (livers were from cadaveric donors who voluntarily donated). No organs were obtained from executed prisoners. Post-operatively, all the patients received standard post-liver transplantation care in the intensive care unit. They received the same immunosuppressive therapy with tacrolimus, daclizumab, mycophenolate mofetil, and methylprednisolone. Short-term tumor recurrences (any recurrence during the first year) of HCC were assessed by measurements of AFP and computed tomography (CT)/magnetic resonance imaging of the chest and abdomen according to a well-defined protocol (ie, every 3 months). As the control group, we also randomly selected 20 patients (15 men, 5 women; mean age: 46 years, age range: 30-65 years) without HCC who underwent OLT due to chronic HBV or HCV cirrhosis as control group during the same period and 20 healthy volunteers (15 men, 5 women; mean age: 43 years, age range: 27-60 years) who had no sign of hepatitis or liver functional abnormality. There were no statistically significant differences in age and sex between the study groups.

**Table 1:** Patients' clinical features

**Clinical features**	
**Gender (male/female)**	27/2
**Age (year) [Mean (rang)]**	48 (37–62)
**Child-Pugh score [No (%)]**	
**A**	14 (48%)
**B + C**	15 (52%)
**TNM stage [No. (%)]**	
**I - II**	12 (41%)
**III - IVa**	17 (59%)
**Milan criteria [No. (%)]**	
**Within**	14 (48%)
**Beyond**	15 (52%)
**Tumor size [No. (%)]**	
**< 5 cm**	16 (55%)
**≥ 5 cm**	13 (45%)
**Tumor number [No. (%)]**	
**< 3**	18 (62%)
**≥ 3**	11 (38%)
**Vascular invasion [No. (%)]**	
**Positive**	15 (52%)
**Negative**	14 (48%)
**Pre-operative locoregional treatment [No. (%)]**	
**No**	17 (59%)
**Yes**	12 (41%)
**Serum AFP [No. (%)]**	
**≤ 20 μg/L**	10 (34%)
**> 20 μg/L**	19 (66%)

The present study was approved by the Institutional Ethics Committee of our institute. The informed consent was obtained from each patient. The procedure met all applicable institutional guidelines of our institute, and governmental regulations concerning the ethical use of donated organs. PB (15 mL) was drawn with a sterile syringe containing ethylenediaminetetraacetic acid (EDTA). PB samples were obtained at the following time points: pre-operatively (just before the skin incision), intra-operatively (during the anhepatic period), and post-operatively (the first week, the first month, and the second month after the operation) from all 29 patients. AFP mRNA detectable each time after operation was considered as "real post-operative AFP mRNA positivity." In the meantime, the control group who provided informed consent were also tested for AFP mRNA, and GPC3 mRNA.

### RNA extraction and real-time RT-PCR

Total RNA was extracted with PB mononuclear cells (PBMCs) with TRIzol reagent (GIBCO-BRL, USA). The real-time RT-PCR used for the detection of AFP mRNA was performed as described previously [11]. The sequences of primers and TaqMan probe of GPC3 were: forward primer (5'-AGAGGCCTTTGAAATTGT-3'), reverse primer (5'-AAATACTTTCAGGTCACGTC-3'), and probe (5'-FAM-ATGCCAAGAACTACA CCAATGC-TAMRA-3'). The conditions for every PCR reaction were 15 min at 95°C, followed by 40 cycles of denaturation for 20 seconds at 95°C and annealing/extension for 60 seconds at 60°C. Data were analyzed with Sequence Detection Software. The level of expression was calculated using the formula [18]:

Relative expression = copy number of target molecule/Copy number of ß-zactin

The RT-PCR assay was repeated twice and performed with an ABI Prism GeneAmp 7300 Sequence Detection System (Applied Biosystems, Foster City, CA)

### Statistical analysis

Kaplan-Meier survival analysis and log-rank test were used to derive the survival curve. To assess the risk factors, univariate and multivariate analyses were performed using the Kaplan-Meier method (long-rank test) and Cox's proportional hazards model. Comparison between two groups was made by independent Student's t test for numerical variables, and x(2) test for categorical variables. One-way ANOVA with Bonferroni post hoc test was used for comparisons between more than two groups. A p value < 0.05 was considered statistically significant. Analyses were made using SPSS® software ver 12 for Windows® (SPSS, Chicago, IL, USA).

## Results

All patients had at least one year of follow-up. HCC recurred in 7 (24%) of 29 patients at 4-11 (median 6.8) months after OLT during the follow-up period. AFP mRNA was positive in 8 (28%) patients pre-operatively, in 15 (52%) intra-operatively, and in 9 (31%) post-operatively. AFP mRNA was positive pre-operatively in 6 (21%) patients-pre-, intra- and post-operatively. None of healthy volunteers had AFP mRNA-expressing cells in their PB, and one (5%) of patients with chronic HBV or HCV cirrhosis without HCC, had AFP mRNA-expressing cells in his PB pre-operatively; these frequencies were significantly different from pre-operative patients with HCC (p = 0.013, p = 0.030, respectively). Of the nine variables assessed, four were significantly related to recurrence in univariate analysis. These were TNM stages, vascular invasion, pre-operative AFP mRNA, and post-operative AFP mRNA (Table 2).

**Table 2:** Univariate analysis in patients with recurrence

**Variables**	**Recurrence **(n/No.) (%)	**p-value a**
**Child-Pugh grading**		0.806
**A**	3/14 (21)	
**B+C**	4/15 (27)	
**TNM stage**		0.013
**I - II**	0/12 (0)	
**III - IVa**	7/17 (41)	
**Milan criteria**		0.244
**Within**	2/14 (14)	
**Beyond**	5/15 (33)	
**Tumor diameter**		0.901
**< 5 cm**	4/16 (25)	
**≥ 5 cm**	3/13 (23)	
**Tumor number**		0.891
**< 3**	4/18 (22)	
**≥ 3**	3/11 (27)	
**Vascular invasion**		0.008
**Positive**	7/16 (44)	
**Negative**	0/13 (0)	
**Pre-operative locoregional treatment**		0.462
**No**	5/17 (29)	
**Yes**	2/12 (17)	
**Serum AFP**		0.188
**≤ 20 μg/L**	1/10 (10)	
**> 20 μg/L**	6/19 (32)	
**Pre-operative AFP mRNA**		0.000
**Negative**	1/21 (5)	
**Positive**	6/8 (75)	
**Postoperative AFP mRNA**		0.000
**Negative**	0/20 (0)	
**Positive**	7/9 (78)	

^a^ p-value from Cox proportional hazards regression analysis.

**Figure 1:**
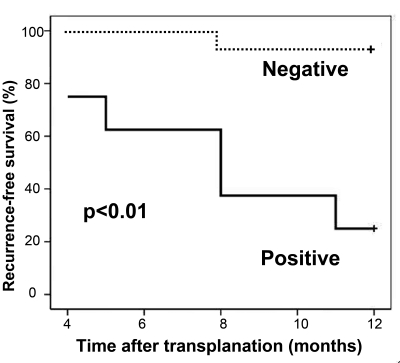
Recurrence-free survival of those with negative pre-operative AFP mRNA was higher than those with positive pre-operative AFP mRNA (Kaplan-Meier method)

The presence of pre-operative AFP mRNA-positive cells in PB was in favor of a lower recurrence-free survival rate (Figure 1). Pre-operative detection of AFP mRNA was found as an independent risk factor for HCC recurrence by multivariate analysis using Cox's proportional hazards model (Table 3). GPC3 mRNA was expressed in all PB samples. There was no significant difference in expression levels of GPC3 mRNA between the HCC group (7.9 ± 3.4×10-3) and healthy volunteers (6.1 ± 2.8×10-3) (p = 0.056), and between patients with chronic viral hepatitis and cirrhosis (6.7 ± 2.9×10-3) (p = 0.214). Of those patients with (6.0 ± 2.5×10-3) and without (6.2 ± 2.6×10-3) tumor recurrence after OLT, there was also no significant difference in GPC3 mRNA expression values (p = 0.858).

**Table 3:** Multivariate analysis in those with recurrence

**Variables**	**x (2)**	**p-value a**	**Relative Risk **(95% CI)
**TNM stages (I - II vs III - IVa )**	0.255	0.614	1.288 (0.483–3.436)
**Vascular invasion (positive vs negative)**	0.209	0.647	1.202 (0.546–2.647)
**Pre-operative AFP mRNA (positive vs negative)**	4.554	0.033	2.909 (1.091–7.759)
**Post-operative AFP mRNA (positive vs negative)**	3.291	0.070	2.619 (0.926–7.410)

^a^ p-value from Cox proportional hazards regression analysis.

## Discussion

It is a well-developed and mature technique in recent years that marked RNA of tumor cells in PB was amplified using RT-PCR to confirm the presence of tumor cells in blood[9][10]. Previous studies showed that AFP mRNA may be investigated as a surrogate marker for isolated tumor cells (ITC) of HCC, and may predict HCC recurrence after curative hepatectomy[19][20]. However, the specificity and prognostic value of AFP mRNA for circulating HCC tumor cells remains questionable[21]. Furthermore, the clinical significance of this molecular technique remains controversial in patients for prediction of HCC recurrence after LT. A recent study by Marubashi, et al, showed that the detection of AFP mRNA in PB pre-operatively could be associated with the HCC recurrence and might serve as a useful predictor for the HCC recurrence in liver transplant patients[22]. Whereas Cheung, et al, showed that plasma AFP mRNA level revealed insignificant association with the HCC recurrence after LT[23]. A higher risk of HCC recurrence in patients with pre-operative AFP mRNA or in patients with AFP mRNA persistently positive pre-operatively has been reported in the literature[23][24]. This study showed that the positive expression rate of AFP mRNA was 28% (8/29) in PB samples pre-operatively. Several literature reports showed that the positive rate of pre-operative AFP mRNA was 20%-50% in hepatic resection and transplantation[24][25][26][27]. This variability can be attributed to in vitro instability of mRNA, differences concerning laboratory techniques, primer selection, time between sample collection and processing, and different patient populations. Pre-operative presence of AFP mRNA in PB may represent shedding of cells from primary HCC; another possibility is the presence of unfound occult micrometastasis that was undetectable pre-operatively.

Operative manipulation would enhance the dissemination of HCC cells into PB; this cell spread could be partly responsible for tumor recurrence after OLT, although this is an intermittent and transient phenomenon. However, it has been shown that surgical intervention induces a release of non-neoplastic liver cells into the circulation and increases false-positive signals of AFP mRNA. To reduce the likelihood of false-positive results, the post-operative blood samples were taken one week after transplantation, as liberated non-neoplastic cells are presumably filtered from the PB within one week, as previously proposed by Ijichi, et al[24]. Our study showed that consistent positive results of AFP mRNA (three times test after operation) should be considered a real post-operative positive result. One possible explanation for post-operative positive result is that a proportion of cancerous cells released from the surgical procedure would still stay in the PB for more than one week in an immunosuppressed patient. Another possibility would be the presence of unresected occult metastases that left undetectable at the time of surgery. The overall recurrence rate in our study was 24% during the first year post-OLT, which is in keeping with rates reported in other studies[28][29]. According to the results of both univariate and multivariate analyses, only pre-operative presence of AFP mRNA was an independent risk factor for HCC recurrence. This test would help to identify patients who may benefit from more intensive post-LT surveillance, altered immunosuppressive regimen or adjuvant chemotherapy. GPC3 expression is frequently increased in HCC[30]. In fact, GPC3 may be reactivated in HCC as frequently as AFP, which has been used extensively as a marker of this cancer. Moreover, GPC3 is reactivated more often than AFP in small dysplastic liver nodules and therefore may be a more reliable early diagnostic marker than AFP[31][32][33][34]. Our results showed that GPC3 mRNA was generally expressed in PB which is coincident with previous studies indicating that GPC3 was widely expressed in tissues and plasma[35][36]. However, we neither detected any significant difference in GPC3 mRNA expression between the HCC and control groups, nor have we found any relationship between the GPC3 expression in PB cells and the tumor recurrence after OLT which suggested that GPC3 mRNA test in PB does not have the same diagnostic value as that of measurement of GPC3 protein level in plasma[16][17][36]. In conclusion, AFP mRNA test should be considered in the pre-operative workup to adequately select suitable patients and the most relevant therapeutic option; evaluation of AFP mRNA may provide important clues for selecting those patients who need adjuvant chemotherapy and intensive follow-up after OLT. But, we should admit that AFP mRNA is only recommended as complementary tests to the conventionally diagnostic methods used. Future studies with higher numbers of patients and longer follow-up need to confirm the clinical usefulness of this assay.
